# Correspondence

**Published:** 1876-11

**Authors:** 


### Correspondence.

[We publish the following correspondence in addition to that upon the same subject in our issue of September, notwithstanding the great demands upon our space, at the request of Prof. Dunster, who asks it as a matter of justice to himself. We do not think our friends or the profession are by this correspondence any nearer an agreement as to the questions at issue, and we must decline to print anything further on the subject.—Eds.]

University of Michigan,
Ann Arbor, Mich., July 22, 1876.

*Prof. Moses Gunn, M.D.*

My Dear Sir: I have the honor to acknowledge the receipt of your communication of the 20th inst. Again I beg leave to intimate that you have evaded my inquiry. I do not desire the opinion of Rush College as to whether we are educating and making homœopathic doctors. We feel quite competent to determine that question ourselves. But we cannot ascertain from the wording of the Rush Medical College resolution or from your letters what is meant by the term, "at that institution." Permit me to correct your assertion that homœopathy is taught by the old and still "regular" medical department of the University of Michigan. This is in no sense whatever true.

I am very respectfully and cheerfully yours.

E. S. DUNSTER, *Acting Dean.*

CHICAGO, July 25, 1876.

*Prof. E. S. Dunster, M.D.*

MY DEAR DOCTOR: You will, perhaps, permit others to entertain the opinion that, in effect, homœopathy is taught by your medical department, for without the co-effort of that department there would be no complete and efficient teaching of homœopathic students in the University of Michigan. You will also, perhaps, permit us to entertain the opinion that the irregular practice of the medical department in this respect, *makes it irregular.*

Now, my dear Doctor, notwithstanding your own "competence" and contempt of our opinion, let me *personally* express the hope that the Regents will use the whole of that appropriation, and more too, if necessary, in equipping a complete and independent department of Homœopathy, and thus relieve your department of the bad odor which attaches to it. I assisted in the organization of the Medical Department, and gave seventeen years hard and honest labor to it; in it I made whatever of reputation I have, and I love it, but only in its original purity. Therefore, notwithstanding your own "cheerfulness," I am, sorrowingly yours.

MOSES GUNN.

UNIVERSITY OF MICHIGAN,
ANN ARBOR, Mich., July 28, 1876.

*Prof. Moses Gunn, M.D.*

MY DEAR SIR: Of course you are entitled to entertain such opinions as you may choose. But let me remind you that opinions do not alter facts. Now the fact is that we are not teaching homœopathy in the old department of Medicine and Surgery any more than you are teaching it in Rush College. Our teaching, like yours, is flatly and diametrically opposed to homœopathy and all one-sided and untrue systems of medicine, and no one probably knows this better than yourself, who has such an intimate acquaintance with the organization and *personnel* of this school. The slip in your logic is that you have made the teaching of homœopathic students identical with the teaching of homœopathy; whereas there is neither identity, similarity nor analogy even between the two. Now, believing as I do religiously, that the best and speediest way of overcoming error is through the teaching of truth, I am still cheerful under the situation. My only sorrow is that so many who claim to be our friends, and to love the institution, will so persistently misrepresent our case. It is much easier, my dear Doctor, to reform a sinner, (and I know that very many in the profession look upon us here as about the vilest of that class,) by convincing him of the error of his way, rather than by charging him with offenses which he has never committed.

I should be most happy to enter into a consideration of the merits of this question — to which you show a tendency in your last note — but time does not permit. Hoping that I may some day have an opportunity of doing so in person,

I am very respectfully, but still cheerfully yours.

E. S. DUNSTER.

[To this note I have received no reply. E. S. D.]

---

SAN FRANCISCO, CAL., Sept. 6, 1876.

EDITOR CHICAGO MEDICAL JOURNAL AND EXAMINER—*Dear Sir:* Thanks to our salubrious climate, our bracing and invigorating sea winds, with a temperature rarely above 70° during the summer months, bowel affections of young children and like diseases do not play as conspicuous parts in our mortality, as they do in your city. Notwithstanding, however, the absence of this class of diseases, the profession have had no reason to complain this year of little to do, as small-pox has existed in an epidemic form in this city since last May. As a matter of course, the Chinese population are accused of having introduced the disease, for it is customary here to charge them as the immediate cause of every occurrence of an unpleasant character, be it an epidemic, an earthquake, or the usual "hard times." Whether this be deserved or undeserved the writer will not undertake to decide. * * * The total number of cases of small-pox reported to the Health office since the outbreak of the epidemic to date is 700; the deaths amount to 180 in all, or over twenty-five per cent. On an average about seventy-five new cases are still reported during each week. To illustrate the criminal intent of very many people to hide from the proper authorities the existence of small-pox in their families, to which in a great measure the continuance of the epidemic is due, we add the following case, which occurred last week: The death of a child in the family of a tailor having been reported to the health officer, this gentleman paid the house a visit. He found the tailor at work in a front room, while in a rear apartment, separated from the workshop by a thin wooden partition, were six children afflicted with small-pox, the seventh having died of the disease. These children had been sick six weeks, and during this time the tailor attended to his business, serving his customers in this atmosphere of infection. Adjoining the shop are, on one side a restaurant, on the other a bakery. That the epidemic has not made frightful ravages under such conditions is solely due to the vigilance of the Board of Health, which latter enjoys in this city (as an exception) unlimited authority in all sanitary matters. The Board is composed of the Mayor and four physicians, selected by reason of their eminence in the profession, and is entirely removed from political influence. It will no doubt interest the many friends of Dr. Cornelius Herz, late Sanitary Inspector in Chicago, to learn that Dr. H. is one of the members of the Board of Health. Shortly after the outbreak of the epidemic a system of "wholesale" vaccination was introduced at the suggestion of Dr. Herz. Physicians were appointed in all sections of the city, whose duty consisted in vaccinating those who applied, free of charge. To remove any possible objection to vaccination, only fresh bovine vaccine virus was used in the majority of cases. Other precautions which were adopted to prevent the spread of the epidemic consisted in the compulsory re-vaccination of all pupils attending the public schools; the vaccination of the crews of every outgoing and incoming vessel; strict enforcement of the quarantine laws; the appointment of additional sanitary inspectors, etc. Owing to these means, the epidemic has been confined to very narrow limits, the number of deaths not averaging over fifteen a week. It must also be remembered that in this densely settled city, with a population of 300,000, there are over 20,000 Chinese, who persist in living in open defiance of any and every sanitary law, all endeavors to the contrary notwithstanding.

Our State Boards of Medical Examiners are at work examining diplomas and issuing licenses to those who have none, but can pass the required examination, in accordance with an act passed by the last Legislature. As this act was so modified as to allow every State Medical Society to appoint a State Board of Examiners, no matter to what system of quackery it might belong to; and as already four such Boards are in existence, it does not require a profound philosophical turn of mind to percieve that a quack need not experience great difficulty in passing the examination required by a State Board of Quacks. If no such law had been passed, the condition of things would have been an improvement on the present system, which to our way of thinking will not accomplish much more than to legalize quackery. The plan adopted by the Medico-Historical Society of Chicago, in publishing an annual Medical Register, is the best way of protecting regular physicians until our legislatures shall be endowed with sufficient common sense to appoint State Boards of Medical Examiners, not composed of charlatans and quacks, but of regular physicians, as is the custom in civilized nations; the United States, however, being an exception.

Yours very truly, EDMUND J. DOERING, M.D.

---

TO THE EDITOR OF THE CHICAGO MEDICAL JOURNAL AND EXAMINER—*Dear Sir:* I wish to call the attention of the profession to the use of Iodine in combination with the Basilicon Ointment for external applications to any part where the use of Iodine would be indicated.

I sought a vehicle which would hold the Iodine when applied, and that would neither irritate or vesicate until it was absorbed. I had used the resin cerate upon pledgets of cotton as a dressing applied to the cervix uteri after the use of the nitrate of silver, when it occurred to me that that was the article for the Iodine.

I asked Mr. Walter Clarke, at 336 West Madison Street, to prepare it. I mix

| | | |
|---|---|---|
| ℞ | Iodin. (Crys) | grs. x. |
| | Potass. Iodid | q. s. |
| | Cerat. Resin | ℥ j. |

M. and use as directed.

I have used it in various ways; the most successful of which is as an application to the cervix uteri when hypertrophied and congested; it is also useful in endocervicitis, or endometritis—in pelvic cellulitis and chronic pneumonia or bronchitis.

For endocervicitis I use the cotton upon the applicator bathed well with the cerate and leave it in the cavity by shoving it off the applicator, a long twist of cotton having been wound about the handle of the applicator and being left in the vagina. A pledget of cotton well covered with the cerate and having a string attached, is next placed against the uterus and the whole is left for from three to twelve hours. When the vaginal cotton is removed, the cervical is also. I consider it a far better application than the pinus canadensis.

In cases of severe endometritis and induration I have used this as the only medication, after a few applications of the nitrate of silver, or the acids, and effected a cure sooner than with any other article.

With this strength I have known it to vesicate but once, and that was after vesication had been induced in the same locality by a severe topical medication, and the ointment had been applied afterward before the parts had healed.

If others think well enough of it to try its use, and report, we would feel greatly obliged. Most respectfully,

MARY HARRIS THOMPSON, M.D.

---

PILES—CARBOLIC ACID TREATMENT.

In calling the attention of the profession to this treatment of hæmorrhoids, it is done feeling that while some of us have known of its virtues for several years, there are many who have not, and whose minds should be attracted to its importance. It is probably safe to say that one in twenty adults have been afflicted with piles. Nor is this to be wondered at when we call to mind the arrangement of the intricate plexus of veins in the submucous tissue which are affected—instead of the hæmorrhoidal veins proper. Were it not for the partial relief by the middle hæmorrhoidal emptying into the internal iliac, when stasis is produced through the superior by portal engorgement, piles would be more frequent.

The cure is effected by causing partial or complete obliteration of the submucous vessels injected, and giving greater firmness to the tissues. The treatment is very satisfactory, producing little pain and rarely any subsequent inflammation. The good effects are permanent, and generally in two or three sittings, two to four days intervening between each visit.

If the patient has not been obliged to stop business before application, it is not necessary during treatment. It is preferable to employ it after the bowels have moved in the morning. If the bowels are in a soluble condition no laxative is needed. The acid used is dissolved in from one to four parts of glycerine, and a portion of morphia or belladonna added, suitable to the age and susceptibility of the patient. From one drop to a drachm of the mixture may be used at one sitting, and repeated every three to six days.

To apply it to longitudinal or internal piles it is necessary for the patient to bend over a chair or bed, or kneel with face to the floor and strain, while an assistant with thumbs and fingers turns out the gut. The tumid veins are made prominent and the medicine injected hypodermically, using a few drops to each vein or pouch. If the veins are very large two or three only should be injected at one sitting. The external tumors are treated by simply injecting a few drops into each. The ulcers are treated by application with camel's hair brush, or simply injecting into the adjacent areolar tissue. If the brush is used a little care must be used not to load the brush and permit the acid to run down the scrotum or thighs.

It is scarcely necessary to say that the treatment is intended for simple cases. If fistula, syphilis, or fissure exist, they need appropriate treatment, and the hæmorrhoidal tumor covering the lower part of the crack in fissure, will always be recognized by the experienced eye from its little cherry-like appearance. If there should be any subsequent inflammation a flax or bran poultice with hops will give relief.

The above treatment is as good as, if not identical with that of Dr. C. H. David and a few others who are selling the right to treat cases, and making the business a specialty.

Very cordially yours, JNO. J. TAYLOR.

Streator, La Salle Co., Ill., July 15, 1876.

### Correspondence.

[The following was received too late for insertion in the proper place.]

Chicago, October 18, 1876.

To the Editor of the Chicago Medical Journal and Examiner — *Dear Sir:* Will you kindly afford me space in your journal for a personal purpose?

In the June number of the Journal and Examiner for the present year there appeared an article written by Dr. A. B. Newkirk, of Hyde Park, Ill., entitled, "A Case of Vesico-Vaginal Fistula and Laceration of the Anterior Perineum," etc., in which my name is mentioned in such a connection as to produce an erroneous impression as to my views and opinions in regard to the case. This impression I desire, through your courtesy, to correct.

The writer of the article referred to, after detailing the history of the case, states that he found, on examination, a vesico-vaginal fistula, a closure of the urethra, several ulcerated surfaces on the vagina, and a laceration of the perineum. He next proceeds to give his conclusions as to the cause of these lesions, attributing them to "the maladroit use of forceps" by a "Dr. J. (homœopathist)," who had attended the patient in her last confinement, six months previously. He then says, "A. H. Mann, M.D., of South Chicago, J. E. Morrison, M.D., of Hyde Park, J. Ramsay Flood, M.D., Assistant Physician of the Woman's Hospital of the State of Illinois, and A. Reeves Jackson, M.D., Surgeon-in-Chief of the same institution, were called in consultation and made a careful examination of the case; *and fully confirmed the views above expressed by myself as to the nature and probable cause of the existing lesions*," etc. (The italics are mine.)

Inasmuch as this passage does not fairly and candidly represent what took place at the only consultation at which I was present, and supposing that the erroneous character of the statement was the result of either forgetfulness or inadvertence on the part of Dr. Newkirk, I wrote him, requesting him to make the proper correction. To my surprise, he declined doing this act of simple justice, and I am therefore impelled to correct his misstatements and omissions myself.

I visited Mrs. Hague, the subject of Dr. Newkirk's report, on the 28th day of January last, for the sole purpose of aiding in the decision of the question as to the propriety of a surgical operation for a vesico-vaginal fistula. The only physicians present were Drs. Newkirk, Flood and myself. The patient was extremely anæmic, and so feeble that it was necessary for others to lift her from one part of the room to another. On examination I found a vesico-vaginal fistula and a laceration of the perineum, as stated in Dr. Newkirk's report. *I also found the os and cervix uteri extensively destroyed by ulceration* — as *not* stated in the report. In addition, the body of the uterus, the cervix (and especially its vaginal portion) were enlarged, and the organ was firmly adherent to the surrounding structures. The extreme upper part of the vagina, as well as the supra-vaginal portion of the cervix uteri, were the seat of marked induration. An abundant watery discharge, tinged with blood and horribly fetid, exuded from the os uteri and the visible ulcerated surface. This disease of the womb I believed, and stated at the time, and still believe, to be cancerous. This opinion was concurred in by Dr. Flood, Dr. Newkirk dissenting. We all agreed that any operation for the cure of the fistula would be highly improper. I fully coincided with the opinion expressed by Dr. Newkirk during the consultation, to the effect that the fistula of the bladder and the rupture of the perineum were not the result of the uterine disease; *but I expressed no opinion as to how or when or by whom these conditions had been produced.*

To my mind, the important feature of the case was the existence of a malignant disease of the uterus, from which the patient was dying, and from which she soon afterwards did die. To the mind of Dr. Newkirk, however, this was of so little consequence that the fact of the existence of any uterine disease whatever is not even mentioned. On the contrary, to him it seems only important to show, and to prove by others, that "Dr. J. (homœopathist)" used the forceps maladroitly.

Yours, respectfully, A. Reeves Jackson.

---

[Owing to an error this letter could not appear in its proper place.]

### THE ARKANSAS STATE MEDICAL ASSOCIATION, AND THE "JUDICIAL COUNCIL" OF THE AMERICAN MEDICAL ASSOCIATION.

To the Editor: I am the president of the Arkansas State Medical Association, a society which for five consecutive years has been officially recognized by the American Medical Association. Its right to such recognition was called in question before the "Judicial Council," at Louisville, in May, 1875. At this meeting of the American Medical Association, the "Council" fully sustained the Arkansas Association, and its delegates were accorded the right of registration. The following extracts from the published "report" of a delegate from Arkansas, embraces, with other matters of interest, the decision of the "Council":

"We, Dr. Thomas Smith and myself, arrived at Louisville on Monday, at 8 o'clock in the evening, and at once repaired to the 'Galt House,' where we understood a large number of the delegates to be assembled, together with the officers of the Association. On presenting ourselves to the permanent Secretary, with credentials, for registration, we were informed by him, and also by the Chairman of Committee on Credentials, that as there was considerable trouble in the medical profession in Arkansas, it had already been decided as best not to admit any of the delegates from that State, but that the whole question would be submitted to the 'Judicial Council' and that a report upon the subject would be presented at that meeting of the American Medical Association.

"To this proposition we at first strenuously objected, claiming that we had no case before the American Medical Association; that we came not for the purpose of making war upon the other delegation who were members of county societies who had severed their connection with the Arkansas State Association, and proposed to interfere with them in no way, notwithstanding the fact that by their hasty action in withdrawing from the State Association, they had cut themselves off from the right of appeal; but simply to represent the Societies whose Delegates we claimed to be.

"Having confidence in the justice of our cause, in the integrity of the gentlemen composing the 'Judicial Council,' and as the two gentlemen named remained firm in their purpose, we finally acceeded to the proposition that the matter should be referred to the 'Council' for adjudication, believing that our case could withstand the scrutiny of fair minded men, and with the hope, also, that a decision coming from a higher body than the State Association might be the means of reconciliation of all differences, and be accepted by dissenting members as a finality. * * * * * *

"At a late hour that evening I again presented myself to the Secretary, offering initiation fees both for Dr. Smith and myself, and demanded registration. This priviledge was denied us, on the ground that objections had been raised against our admission; and the excuse offered when asked why the other Delegates had been allowed to register when we had been given assurances to the contrary, was that '*he had to register them because no objections had been offered against them.*'

"I desire to call the especial attention of the Society to this part of my report, that it may become a matter of record that, on the authority of the permanent Secretary, no objection or opposition was made to other medical gentlemen of this State claiming the rights of Delegates until we, ourselves, had been protested out of representation.

"I then inquired of the Secretary if I possessed the right to enter objections. He informed me that I did—'that any one could do so.' I then, very hurriedly, as the Secretary was about to close his books, wrote off and filed a protest, but as I was unable to make a copy, I will here reproduce it from memory, as correctly as possible. To this protest I signed Dr. Smith's name, which action on my part afterward received his hearty aproval, viz.:

"'We, the undersigned, Delegates from the 'Arkansas State Medical Association' and 'Little Rock and Pulaski County Medical Society,' do hereby enter our solemn protest against the reception of the gentlemen named as Delegates to the 'American Medical Association,' namely: Dr. P. O. Hooper, 'College of Physicians and Surgeons,' Little Rock; Dr. J. H. Lenow, 'College of Physicians and Surgeons,' Little Rock; Dr. D. A. Linthicum, 'Medical Institute,' Phillips County, for the following reasons, to-wit.:

"'*First*—Said gentlemen have publicly withdrawn from the 'Arkansas State Medical Association.'

"'*Second*—The local societies which they represent have adopted resolutions denouncing and repudiating the said 'State Medical Association' and refusing to affiliate with any who sustain the action of a majority of that body.

"'*Third*—These resolutions were published in both the medical and secular press throughout the country.

"'*Fourth*—The 'plan of organization' of the 'American Medical Association' provides 'that members shall consist of Delegates from permanently organized County Societies, and such District Societies as are represented in the State Society,' etc.

"'*Fifth*—We claim that these gentlemen having severed their connection with said State Society, and being no longer represented therein, they are not, under the Constitution, entitled to representation in the 'American Medical Association.'

(Signed,) "'Thomas Smith, M.D.,
"'J. A. Dibrell, Jr., M.D.'

"A consultation among the gentlemen hereinbefore mentioned seemed to have been held over this paper, for I was soon informed by several gentlemen, including the Secretary, that *I* would be allowed to register as Delegate from my local Society, but that Dr. Smith, from the State Society, would be excluded. This proposition I emphatically declined, for obvious reasons, and so matters stood until the next day—the first of the meeting—when the Secretary read off the names of three or four hundred Delegates and permanent members, including those of Drs. J. H. Lenow, P. O. Hooper and D. A. Linthicum, but omiting Dr. Smith's name, and also my own.

"After the reading was concluded, the Chairman of the Committee on Credentials arose and said that the Delegates from the 'Arkansas State Medical Association' were rejected, and made a motion that the matter be referred to the 'Judicial Council,' which motion was adopted.

"From this time until Wednesday, night at 11 o'clock, we were five times summoned to appear before the 'Judicial Council,' and on each occasion waited for hours without being once called into its presence. At this hour we were informed that the Arkansas question had been decided, and that our evidence was not required, as no case had been made against the 'Arkansas State Medical Association.' On Thursday the 'Judicial Council' presented a report, from which the following is an extract, viz.:

"'*Resolved*, That the Delegates from said State Medical Association (Arkansas) should be admitted to proper registration at this meeting of the 'American Medical Association;' also, that the protest of the local Societies of Arkansas be referred back to their State Society for adjudication.' * * *

"Respectfully submitted,

"J. A. DIBRELL, JR., M.D."

Little Rock, Ark., June, 1875.

The "trouble in the medical profession in Arkansas" arose from a difference of opinion concerning the eligibility of an applicant for membership in the Arkansas State Medical Association. This applicant, in strict conformity with the rules of the Association, was finally admitted to membership, when the members who had opposed his admission withdrew in a body. From this dissatisfied minority sprung the "objections" to the delegates representing the State Medical Association of Arkansas, at Louisville, in May, 1875.

At that meeting of the American Medical Association, the "Judicial Council" rightfully assumed that it was beyond its province to in any manner consider the *merits* of the question involving dissatisfaction in medical circles in Arkansas, but was bound by every rule of right to endorse the action of the State Medical Association, as against a dissenting minority. Further, that "dissenting minority" was very properly referred to its "State Society" for an adjudication of its questions of grievance.

After the meeting of the American Medical Association, in May, 1875, instead of formally presenting their complaints before the Arkansas State Medical Association, the gentlemen composing the dissatisfied minority adopted the cunning device of organizing what they artfully termed "*The Arkansas State Medical Society;*" thus placing themselves in open hostility to the express direction of the "Judicial Council." Delegates from this so called "State Society" were duly dispatched to Philadelphia, and, so far as is known, without question were allowed registration at the last meeting of the American Medical Association in that city. But when the delegation from the *State Medical Association* of Arkansas asked the priviledge of registration it was denied them. Diligent inquiry failed to elicit any satisfactory explanation of this extraordinary refusal of a hitherto conceded right. They were merely informed that a "protest" against their admission had been placed in the hands of the "Judicial Council" by the delegates from the *Arkansas State Society!* The controversy between the Arkansas State Medical Association and the persons constituting the "*State Society*" had been decided a year before, adversely to the latter, by the "Judicial Council;" when a reversal of its former verdict contemplated the destruction of a State Medical Association, ought not the "Council" to have been able to present something more than a mere "protest" in explanation — especially when that "protest" emanated from known enemies of the Association doomed to annihilation?

The Arkansas State Medical Association, notwithstanding, maintains its organization, and its delegates will present themselves again, demanding registration, at the next meeting of the American Medical Association, in Chicago, and it remains to be seen if its members shall be disfranchised and disgraced without good and sufficient cause being shown.

This is one of the questions that the members of the American Medical Association must be prepared to consider at their next meeting. Respectfully,

W. H. Barry, M.D.

Little Rock, Ark., Sept. 20, 1876.

